# The SCRIPT trial: study protocol for a randomised controlled trial of a polygenic risk score to tailor colorectal cancer screening in primary care

**DOI:** 10.1186/s13063-022-06734-7

**Published:** 2022-09-27

**Authors:** Sibel Saya, Lucy Boyd, Patty Chondros, Mairead McNamara, Michelle King, Shakira Milton, Richard De Abreu Lourenco, Malcolm Clark, George Fishman, Julie Marker, Cheri Ostroff, Richard Allman, Fiona M. Walter, Daniel Buchanan, Ingrid Winship, Jennifer McIntosh, Finlay Macrae, Mark Jenkins, Jon Emery

**Affiliations:** 1 Primary Care Cancer Research Group, Department of General Practice, Centre for Cancer Research, The University of Melbourne, Victorian Comprehensive Cancer Centre, Level 10, 305 Grattan Street, Melbourne, Victoria 3000 Australia; 2 Centre for Cancer Research, University of Melbourne, Melbourne, Australia; 3 Centre for Health Economics Research and Evaluation, University of Technology Sydney, Sydney, Australia; 4 IPN Medical Centres, Camberwell, Australia; 5 Consumer Advisory Group, Primary Care Collaborative Cancer Clinical Trials Group, Carlton, Australia; 6 Centre for Workplace Excellence, University of South Australia, Adelaide, Australia; 7 Genetic Technologies/Phenogen Sciences, Fitzroy, Australia; 8 Centre for Epidemiology and Biostatistics, The University of Melbourne, Melbourne, Australia; 9 Wolfson Institute of Population Health, Barts and The London School of Medicine and Dentistry, Queen Mary University of London, London, UK; 10 Department of Clinical Pathology, University of Melbourne, Melbourne, Australia; 11 Department of Medicine, Melbourne Medical School, University of Melbourne, Melbourne, Australia; 12 Genetic Medicine, Royal Melbourne Hospital, Melbourne, Australia; 13 HumaniSE Lab, Department of Software Systems and Cybersecurity, Monash University, Clayton, Australia; 14 Colorectal Medicine and Genetics, The Royal Melbourne Hospital, Melbourne, Australia

**Keywords:** Colorectal cancer, Cancer screening, Risk-stratified screening, Primary care, Faecal occult Blood test, General practice, Precision screening, Polygenic risk score, Clinical utility genomics

## Abstract

**Background:**

Polygenic risk scores (PRSs) can predict the risk of colorectal cancer (CRC) and target screening more precisely than current guidelines using age and family history alone. Primary care, as a far-reaching point of healthcare and routine provider of cancer screening and risk information, may be an ideal location for their widespread implementation.

**Methods:**

This trial aims to determine whether the SCRIPT intervention results in more risk-appropriate CRC screening after 12 months in individuals attending general practice, compared with standard cancer risk reduction information. The SCRIPT intervention consists of a CRC PRS, tailored risk-specific screening recommendations and a risk report for participants and their GP, delivered in general practice. Patients aged between 45 and 70 inclusive, attending their GP, will be approached for participation. For those over 50, only those overdue for CRC screening will be eligible to participate. Two hundred and seventy-four participants will be randomised to the intervention or control arms, stratified by general practice, using a computer-generated allocation sequence. The primary outcome is risk-appropriate CRC screening after 12 months. For those in the intervention arm, risk-appropriate screening is defined using PRS-derived risk; for those in the control arm, it is defined using family history and national screening guidelines. Timing, type and results of the previous screening are considered in both arms. Objective health service data will capture screening behaviour. Secondary outcomes include cancer-specific worry, risk perception, predictors of CRC screening behaviour, screening intentions and health service use at 1, 6 and 12 months post-intervention delivery.

**Discussion:**

This trial aims to determine whether a PRS-derived personalised CRC risk estimate delivered in primary care increases risk-appropriate CRC screening. A future population risk-stratified CRC screening programme could incorporate risk assessment within primary care while encouraging adherence to targeted screening recommendations.

**Trial registration:**

Australian and New Zealand Clinical Trial Registry ACTRN12621000092897p. Registered on 1 February 2021.

**Supplementary Information:**

The online version contains supplementary material available at 10.1186/s13063-022-06734-7.

## Administrative information

Note: the numbers in curly brackets in this protocol refer to SPIRIT checklist item numbers. The order of the items has been modified to group similar items (see http://www.equator-network.org/reporting-guidelines/spirit-2727-statement-defining-standard-protocol-items-for-clinical-trials/).

**Table:** 

Title {1}	The SCRIPT Trial: a randomised controlled trial of a polygenic risk score to tailor bowel cancer screening in primary care
Trial registration {2a and 2b}	ANZCTR: ACTRN12621000092897p
Protocol version {3}	SCRIPT protocol V1.2 14/02/2022
Funding {4}	Cancer Australia Priority-driven Collaborative Cancer Research Scheme
Author details {5a}	Sibel Saya^1, 2^, Lucy Boyd^1, 2^, Patty Chondros^1^, Mairead McNamara^1, 2^, Michelle King^1, 2^, Shakira Milton^1, 2^, Richard De Abreu Lourenco^3^, Malcolm Clark^4^, George Fishman^5^, Julie Marker^5^, Cheri Ostroff^6^, Richard Allman^7, 8^, Fiona M. Walter^1, 9^, Daniel Buchanan^2, 8, 10^, Ingrid Winship^11, 12^, Jennifer McIntosh^1, 13^, Finlay Macrae^11, 14^, Mark Jenkins^2, 8^, Jon Emery^1, 2^ 1. Department of General Practice, Melbourne Medical School, University of Melbourne, Melbourne, Australia 2. Centre for Cancer Research, University of Melbourne, Melbourne, Australia 3. Centre for Health Economics Research and Evaluation, University of Technology Sydney, Sydney, Australia 4. IPN Medical Centres, Camberwell, Australia 5. Consumer Advisory Group, Primary Care Collaborative Cancer Clinical Trials Group, Carlton, Australia 6. Centre for Workplace Excellence, University of South Australia, Adelaide, Australia 7. Genetic Technologies / Phenogen Sciences, Fitzroy, Australia 8. Centre for Epidemiology and Biostatistics, The University of Melbourne, Melbourne, Australia 9. Wolfson Institute of Population Health, Barts and The London School of Medicine and Dentistry, Queen Mary University of London, United Kingdom 10. Department of Clinical Pathology, University of Melbourne, Melbourne, Australia 11. Department of Medicine, Melbourne Medical School, University of Melbourne, Melbourne, Australia 12. Genetic Medicine, Royal Melbourne Hospital, Melbourne, Australia 13. HumaniSE Lab, Department of Software Systems and Cybersecurity, Monash University, Clayton, Australia 14. Colorectal Medicine and Genetics, The Royal Melbourne Hospital, Melbourne, Australia
Name and contact information for the trial sponsor {5b}	The University of Melbourne, Prof Jon Emery (sponsor-investigator), jon.emery@unimelb.edu.au
Role of sponsor {5c}	The Sponsor does not have input or ultimate authority in the study design; collection, management, analysis, and interpretation of data; writing of the report; and the decision to submit the report for publication.

## Introduction

### Background and rationale {6a}

#### Colorectal cancer

Colorectal cancer (CRC) is the second commonest non-cutaneous malignancy in Australia (15,540 projected cases in 2021) and second only to lung cancer in terms of cancer mortality (5295 projected deaths in 2021) [1]. Early detection is important in terms of both survival (5-year survival: 84% for stage 1, 77% for stage 2, 64% for stage 3 and 19% for stage 4), patient experience and cost of treatment (average costs per diagnosis: A$34,337 for stage 1, A$53,487 for stage 2, A$79,924 for stage 3 and A$71,156 for stage 4) [2]. Over 40% of CRCs are diagnosed at stage 3 or 4 in Australia. Screening and subsequent early detection is an effective method to reduce the mortality from CRC [3].

#### People are not at equal risk of colorectal cancer

While the average lifetime population risk of CRC is around 5% in Australia, there is a wide spectrum of risks [4]. CRC risk for the top quartile of the population is 20 times greater than for those in the lowest quartile; 90% of CRC occurs in those in the upper half of the population for risk [4]. In Australia, the 2017 NHMRC-endorsed Guidelines for the Prevention, Early Detection and Management of Colorectal Cancer [5, 6] recommend a risk-based approach to CRC screening, as do other international guidelines [7]. However, the Australian guidelines rely only on age and family history to determine CRC risk. While family history does impact the risk of CRC, incorporating a complete family history into a CRC risk model gives in an area under the receiver operating curve (AUROC) of up to 0.54 [8, 9]. This indicates that a random person with CRC will have a stronger family history than a random person without CRC on average 54% of the time, meaning family history as a risk predictor performs little better than chance. There are now more precise methods using genomic data to estimate a person’s risk of CRC, which could enable expensive and higher risk preventive strategies such as colonoscopy, to be better targeted to those who will benefit most.

#### The importance of genetic and genomic risk for colorectal cancer

There are well recognised but relatively rare inherited syndromes which significantly increase the risk of CRC, such as Lynch syndrome. Predictive genetic testing for mutations in specific genes can identify individuals with these syndromes, but these inherited syndromes account for approximately only 5% of CRC [10]. Recent genome-wide association studies have identified multiple common single nucleotide polymorphisms (SNPs) which are associated with an increased risk of CRC [11]. These SNPs and their associated cancer risk estimates can be combined to create polygenic risk scores (PRS). A 45 SNP-based PRS provides much greater discrimination than age and family history alone (AUROC 0.71 for the PRS plus family history model) [12]. This PRS has been validated in an external population, demonstrating that this PRS plus simple family history information and age over and under 60 has an AUROC of 0.66 for men and 0.64 for women [8]. If this 45 SNP-derived PRS were applied to stratify CRC screening, people in the top quintile of the PRS with a family history of CRC would commence screening at 41 years, compared to the current standard of 50. However, if they had a family history but were in the lowest quintile of PRS, no additional screening would be required before age 50 [13]. One caveat of the clinical validity and utility of PRSs is that the majority of evidence is established for European populations [14], with reduced accuracy in non-European groups. For this CRC PRS, there is evidence that the PRS discriminated colorectal cancer cases from controls more poorly in a heterogeneous group of non-White/non-Europeans than Europeans [8].

#### Targeted screening and risk

The classical dichotomy in disease prevention is between targeting strategies to individuals at high risk versus the general population. Internationally, there is a growing concern about the relative harms as opposed to the benefits of general population cancer screening programs [15]. Advances in cancer epidemiology and the potential application of genomic testing have led to arguments for a third approach: “a prior assessment of risk, applied to the whole population, followed by the assignment of individuals to a risk stratum and the tailoring of the interventions offered to each group. In so doing, it aims to optimise the benefit-harm ratio and the cost-effectiveness of the public health program” [16].

#### Adherence to screening recommendations

In Australia, population CRC screening is provided through the National Bowel Cancer Screening Program (NBCSP) which sends biennial immunochemical faecal occult blood tests (iFOBTs) to all aged 50–74. However, uptake of iFOBT is only 42%, despite the kit being free and sent directly to those eligible [17]. It has been suggested that individualised health risk information could motivate patients to participate in screening tests when compared to the current standard of providing similar healthy living advice to all [18, 19]. Several meta-analyses of studies seeking to motivate behaviour change after genomic testing for a variety of common conditions have been reported [20, 21]. Across a range of lifestyle behaviours, there were no consistent differences in behaviour between those who received personalised risk results and those who did not. One review has suggested that genomics by itself cannot be expected to alter health behaviours, but it can be used not only to target screening to those who need it most, but to tailor the efforts of health professionals to reduce risky health behaviours to those in higher risk groups [22].

To date, only two studies have examined the effect on CRC screening adherence after a personalised genomic risk test in those without a family history [23, 24]. In the first study, only five of 47 participants were non-adherent to CRC screening at recruitment [23]. The second did not show a positive effect of the genomic test, but it only included one genetic risk factor, giving a small variation in risk [24]. A major factor that may have also influenced the results of these previous studies was the research context in which participants were seen and counselled. Health professional endorsement of interventions has been seen to increase their efficacy, for example, increased uptake of iFOBT screening after a letter from the patient’s GP [25]. It is possible that including GP engagement in such an intervention will encourage more screening adherence.

#### What SCRIPT adds

Ultimately, precision screening aims to target screening based on better estimates of disease risk (e.g. using PRSs) and also to encourage people to adhere to those personalised screening recommendations. The SCRIPT trial aims to test an intervention within primary care that incorporates CRC risk prediction using a PRS, tailored risk-based screening recommendations and risk reports for use by patients and their GP to encourage risk-appropriate screening.

### Objectives {7}

#### Primary objective

The primary objective is to evaluate the impact of the SCRIPT intervention on risk-appropriate CRC screening after 12 months in general practice patients aged 45–70 due or overdue CRC screening, compared with standard cancer prevention information. The SCRIPT intervention comprises a personalised risk estimate derived from a PRS, tailored screening recommendations and risk reports, delivered in primary care.

#### Secondary objectives

The secondary objectives of this study are to determine the impact of the SCRIPT intervention compared with standard cancer prevention information at 1, 6 and 12 months on participants’:

- CRC risk perception
- Cancer-specific anxiety
- Elements known to influence CRC screening behaviour
- Cancer screening intentions
- Health care utilisation

### Trial design {8}

The SCRIPT trial is a multi-site, phase II individually randomised controlled trial [26]. The study will test the implementation of the SCRIPT intervention in primary care, aiming to increase risk-appropriate CRC screening. Participants will be randomly allocated 1:1 to the intervention arm who will receive the SCRIPT intervention or the control arm who will receive standard cancer prevention information (Additional file 1).

In the intervention arm, personalised CRC risk results from the PRS and resulting screening recommendations will be available 2–3 weeks after sample provision, when the researcher will discuss the results with participants. Standard cancer prevention information will be discussed with control arm participants immediately after randomisation. All participants will be followed up via questionnaires at 1, 6 and 12 months and collection of objective data regarding their CRC screening behaviour from their medical records at 12 months. DNA will be collected from all participants at baseline, and the PRS result and screening recommendations will be offered to control participants after the primary endpoint (12 months). Given the nature of the intervention, it is not possible to blind participants to their allocation; however, researchers collecting follow-up data will be blinded.

## Methods: participants, interventions and outcomes

### Study setting {9}

Participants will be recruited from six to ten general practices (depending on size and recruitment rates) across Victoria, Australia. Randomisation will be stratified by general practice site.

### Eligibility criteria {10}

#### Eligibility criteria for clinics

General practices will be included if they are of a size with sufficient volume of potential participants. Individual GPs within clinics will be consented to the study for researchers to approach their patients to participate.

#### Eligibility criteria for participants

Eligible participants will be general practice patients aged 45–70 who have an appointment for any reason within a week of the approach date to see a GP consented to the SCRIPT study, meeting all of the following criteria:

- Are aged between 45 and 70 years inclusive
- Are able to read and write English and competent to give informed consent
- Are contactable over the next 12 months for the study follow-up
- For those aged over 50, report being due for some CRC screening within the next 12 months (e.g. for those with no or minimal family history, according to the NHMRC guidelines [5], have not had an iFOBT within the past year and have not have a colonoscopy within the past 3 years; for those with a moderate family history [5], have not had a colonoscopy within the past 4 years)

This last eligibility criterion was chosen given our experience in a previous study, the CRISP trial, that showed the behavioural impact of personalised risk estimate is greatest in those due screening [27] (results paper in submission). This applies to those aged 50 and over, but not to those aged 45–49 who are not routinely eligible for population CRC screening.

Participants cannot meet any of the following criteria:

- Have been diagnosed with CRC
- Have recent changes to bowel habits, rectal bleeding or a diagnosis of inflammatory bowel disease
- Have a known genetic predisposition to CRC or a family history of cancer that requires referral for assessment of a genetic predisposition to CRC (according to the NHMRC guidelines [5]). This includes:
  - ◦ Those confirmed as carrying a pathogenic mutation in a gene associated with a high-risk familial syndrome
  - ◦ Those with a relative confirmed as carrying a pathogenic mutation in a gene associated with a high-risk familial syndrome, who have not themselves been tested
  - ◦ Those with a relative with multiple CRCs
  - ◦ Those with at least three first-degree or second-degree relatives with a Lynch syndrome-related cancer (colorectal, endometrial, ovarian, stomach, small bowel, renal pelvis or ureter, biliary tract, brain) with at least one diagnosed before age 55 years
- Have a grandparent born in Africa or of African ancestry (see below)

#### Ancestry eligibility criteria

It is a well-acknowledged limitation of PRSs that they were developed from populations predominantly of Caucasian ancestry [28, 29]. Given this, there are significant issues of accuracy of risk estimates and applicability to those of other ethnicities, in particular in those of African ancestry [30]. There are substantial efforts in progress to correct these inequalities, including large international consortia that aim to perform new genome-wide association studies in diverse samples to develop more widely applicable PRSs [31–33]. Clinical harm can arise when an inaccurate risk estimate (due to the application of a PRS to a non-European person) results in the wrong screening recommendation. For example, not screening when they should be and thereby missing early detection of cancer, or screening when they should not be and thereby at risk of an adverse screening event/injury.

Given the risk associated with providing an inaccurate risk estimate to participants who are of African ancestry, the study investigators determined that this final exclusion criterion was necessary. Participants with African ancestry who are excluded from the trial will be offered a brochure outlining the bowel cancer screening available through the NBCSP and the opportunity to complete a CRISP CRC risk assessment in place of being able to participate in the study. The CRISP tool contains a validated lifestyle risk prediction model that is suitable for people of all ethnicities. We have previously utilised this tool in another RCT [27]. The model has been externally validated [34]. The researcher will take the ineligible patient through the CRISP tool (https://crisp.org.au/crisp-clinic) which provides a 10-year-risk of CRC and a screening recommendation for the participant. They will then be recommended to discuss their CRISP results with their GP.

### Who will take informed consent? {26a}

#### Informed consent of GPs

Members of the research team will present interested GP clinics the study rationale and participant recruitment processes, then invite discussion about the study. This includes information about the current NHMRC guidelines for CRC screening, the evidence behind the PRS and its ability to predict the risk of CRC and that they will receive CRC screening recommendations for their patients in the intervention arm within the SCRIPT study. It will be emphasised that they should use their clinical judgement along with the CRC screening recommendations provided within the study. Each GP will be given a GP information sheet about the study, given the opportunity to ask questions and individually consented to the study to allow recruitment of their patients.

#### Informed consent of participants

Trained research assistants will provide individuals who have GP appointments with consented GP verbal and written information about the trial, check their eligibility and answer any questions about the study. A second research assistant will obtain written informed consent if they agree to participate in the trial. Due to COVID-19 and resulting government restrictions, both face-to-face and teletrial methods will be used for approach, eligibility assessment and informed consent discussions with potential participants [35]. Additional and optional written consent will also be sought for release of participants’ NBCSP data via the Australian Institute of Health and Welfare (AIHW) and Medicare Benefits Schedule (MBS) data via Services Australia. Paper copies of all consent forms will be posted to teletrial potential participants and they will only be randomised once they have provided written consent. Two attempts will be made to remind these potential participants to return documents, after which they will be marked as having refused participation and all identifying information destroyed.

#### Additional consent provisions for collection and use of participant data and biological specimens {26b}

Participants will give specific consent for the study, in that their data will not be used for future studies. Any excess DNA will be securely disposed of by the laboratory conducting the genomic test.

## Interventions

### Explanation for the choice of comparators {6b}

Control participants will have a standardised consultation in person or over the telephone, delivered by the second research assistant, during which an adapted version of the Cancer Council Victoria 2018 ‘Cut your cancer risk’ brochure will be discussed (Additional file 1), and a hard copy provided via post or email. The focus of the control consultation will be about how to reduce cancer risk by modifying behaviour, along with brief information about the three national cancer screening programs (biennial iFOBTs for bowel cancer from 50–74, biennial mammogram for breast cancer from 50–74 and cervical screening tests every 5 years from 25–74). This is designed as a credible ‘attention control’ while not altering ‘usual care’ since it is unlikely to prompt discussions about CRC risk or screening with their GP. It also increases engagement in the trial for control participants to minimise attrition.

The control arm will have the option to receive their DNA test results report after the 12-month intervention period from the time of recruitment. This strategy has been employed to increase engagement and participation in the trial. Control participants will be reminded of this option when told of their arm allocation and asked if they want to take this up at the end of the final questionnaire for the study, sent 12 months after recruitment. For those who would like their genomic test results, it will be discussed with them over the phone, a hard copy sent to them via post and a copy placed in their GP record.

### Intervention description {11a}

The SCRIPT intervention is a ‘complex intervention’: that is, it contains several interacting components. The main component is a CRC PRS, with a post-test consultation to discuss the participant’s personal risk of CRC with an associated risk report for the participant and their GP. The reports are designed to encourage risk-appropriate screening and referral behaviours, based on the previous CRISP trial reports [36–38] and SCRIPT pilot work [39].

Participants will attend a consultation, within 1 week either side of their scheduled GP appointment, delivered by a trained research assistant either in person at their GP or via video call, where a brief discussion about the CRC PRS will take place. This will include information about the test and its potential implications, including potential impact on CRC screening recommendations.

A PRS will be generated from the presence or absence of each of the 45 SNPs, using the most up-to-date relative risks of CRC for each DNA variant and also the individual’s family history of CRC. For those who are of East Asian ancestry (e.g. Chinese, Japanese and Korean), there are sufficiently large GWAS studies for ancestry-specific relative risks for CRC for each of the 45 SNPs included in the PRS. Given this, these estimates will be used when calculating a PRS for those who self-identify as East Asian. For other ethnicities, there is evidence that European developed PRSs predict the risk of breast cancer in those of non-European ancestries [40] and in the absence of ancestry-specific PRSs, the European PRS estimates for CRC will be used.

Applying Australian age-sex incidence data to each individual’s PRS, a 10-year absolute risk of CRC will be calculated. Screening recommendations (iFOBT or colonoscopy) will be generated in accordance with the 2017 NHMRC-endorsed national guidelines which are based on 10-year absolute risks of CRC. Those with a 10-year risk of less than 1% are not recommended screening; those with a 10-year risk of 1% or greater and less than 4% will be recommended iFOBT; and those with a 10-year risk of 4% or greater will be recommended colonoscopic screening. However, within the study, no participant will be recommended less screening than the current NHMRC-endorsed national guidelines (for example, if a participant in their 50s has a PRS-derived 10-year risk <1%, they will still be recommended iFOBT screening).

Results from the PRS test will be available approximately 2–3 weeks after their pre-test consultation and sample provision. Participants in the intervention arm will discuss the results of their PRS with the research assistant. They will be asked to make an appointment with their GP directly after the risk result appointment. As a first option, both result discussions will occur in person at the participant’s GP clinic. Given the current COVID-19 pandemic, in the case of lockdown rules preventing study staff from attending the clinic, results will be given using an online meeting tool (e.g. Zoom [41] or doxy.me [42]) with a screensharing facility, allowing for the researcher to show the participant their risk report, prior to posting them a paper copy.

At the meeting, a report summarising the participant’s CRC risk and screening recommendations will be given to them to discuss with their GP. Those who are due screening with an iFOBT will be encouraged to discuss with their GP and either provided an iFOBT kit on the day, or their GP will order one to be sent in the post via the National Cancer Screening Register. GPs will have the final responsibility to organise appropriate CRC screening.

**Fig. 1:**
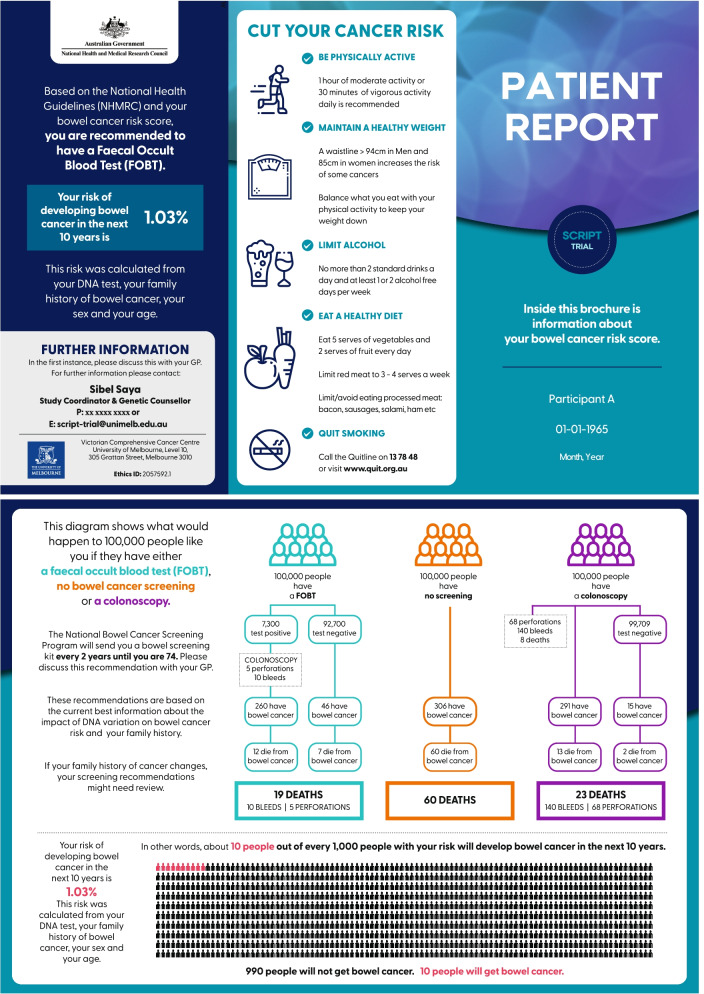
Example colorectal cancer risk report and screening recommendations for a participant at average risk in the SCRIPT study

**Fig. 2:**
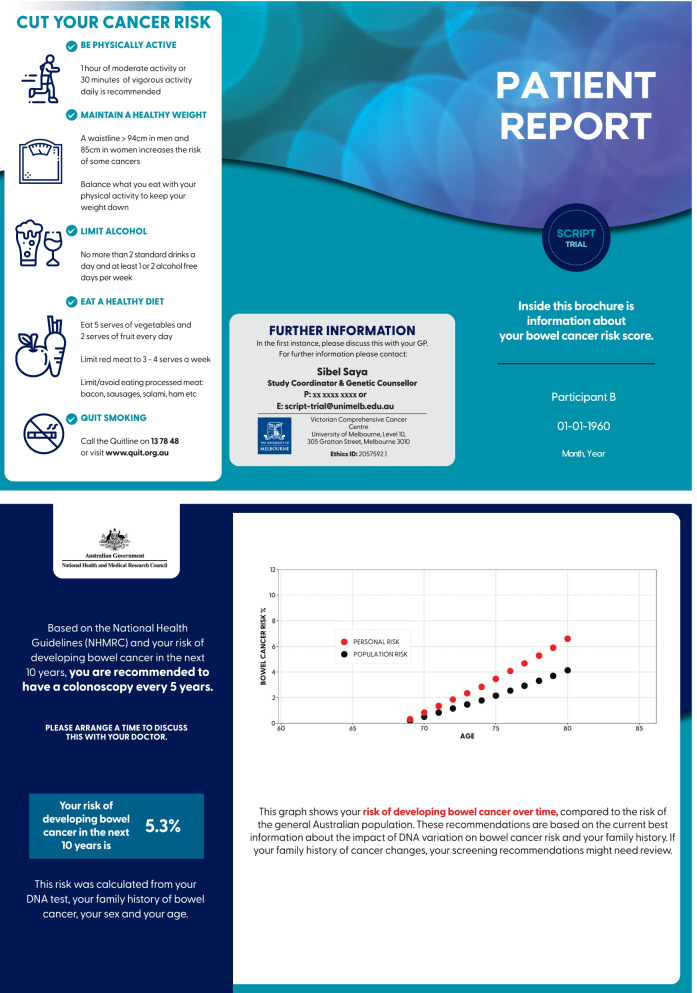
Example colorectal cancer risk report and screening recommendations for a participant at moderate risk in the SCRIPT study

Examples of risk reports can be seen in Figs. 1 and 2. The risk report is designed to increase response efficacy for screening (a person’s belief that the behaviour will reduce their disease risk). A version of the risk report tailored for the GP’s use will also be uploaded to the participant’s GP record (Additional files 2 and 3). All participants recommended an iFOBT from their PRS will be given a brief demonstration on its use via a standard NBCSP video to increase their self-efficacy to perform the test [43]. To encourage adherence with iFOBT, we will send a reminder to complete testing as an SMS message after 1 month to participants in the intervention arm who are due an iFOBT. For those who have reported having polyps found on previous colonoscopy, an information sheet regarding the NHMRC guidelines on surveillance colonoscopy after polypectomy will be given to participants’ GPs to assist their clinical discussion and determine the timing and need for further colonoscopic surveillance.

### Criteria for discontinuing or modifying allocated interventions {11b}

Participants can withdraw from the study at any time without giving a reason, as stated to them prior to informed consent. If this occurs prior to provision of CRC risk results, participants will not receive the full intervention. Participants will be given the choice as to whether their withdrawal will only be from further contact from the trial team or withdrawal of all their unanalysed data.

### Strategies to improve adherence to interventions {11c}

For those who do not attend their scheduled risk result appointment, three attempts will be made to reschedule. After three unsuccessful contacts or three non-attendances at their result appointment, they will be sent their risk results in the post and a copy uploaded to their GP record.

During the initial presentation of the study procedures and purpose with GPs prior to their consent, they will be encouraged to discuss the risk information provided to their patients in the trial and order screening according to their clinical judgement. To reduce the risk of contamination in the control arm, we will encourage each GP to continue their normal practice with patients who do not have a CRC risk report. We will assess potential contamination during the trial by what proportion of participants in the control arm have GP-ordered iFOBTs within 4 weeks of consent.

### Relevant concomitant care permitted or prohibited during the trial {11d}

No alteration to usual care is required during the trial, and therefore, there is no concomitant care that is prohibited.

### Provisions for post-trial care {30}

There are no anticipated harms associated with the intervention in the study. All participants are recommended CRC screening at least as much as what is recommended in the NHMRC guidelines. Additionally, GPs in the study are informed that they should use their clinical judgement along with the personalised CRC screening recommendations; clinical responsibility remains with each participant’s GP. Previous studies returned genomic risk results for cancer have not found a substantial risk of anxiety or psychological harm from knowledge of future risk [44]. Therefore, there are no provisions for post-trial care.

### Outcomes {12}

#### Primary outcome

The primary outcome is the difference between the two arms in the proportion of participants who have had risk-appropriate CRC screening at 12 months’ follow-up. The appropriate screening test in the intervention arm will be determined by the screening recommended according to their absolute 10-year risk of CRC derived from the PRS and family history. For those in the control arm, the appropriate screening test is determined by family history according to the NHMRC guidelines [5].

Risk-appropriateness of CRC screening was chosen as the primary outcome as previous cost-effectiveness studies have shown that CRC population screening targeted using a PRS would be cost-effective if the behavioural effect of returning the risk estimate increased screening by 5% [45]. Additionally, a study modelling the impact of various participation levels in the Australian NBCSP showed that increasing participation from 40 to 60% would result in 24,800 deaths prevented [3].

#### Secondary outcomes

Difference between the SCRIPT intervention arm and standard cancer prevention information arm for the following secondary outcomes:

- Perceived personal risk of CRC, using mean absolute risk estimates and proportions estimating themselves as average, higher than average and lower than average at 1, 6 and 12 months, will be measured using existing validated items [46]
- Mean cancer-specific anxiety at 1, 6 and 12 months on the Cancer Worry Scale [47]
- Mean scores of predictors of CRC screening behaviour (salience and coherence, response efficacy, cancer worries, social influence, self-efficacy) at 1, 6 and 12 months taken from the Preventative Health Model [48]
- Proportions with CRC screening intentions at 1 and 6 months based on the Theory of Planned Behaviour [49]
- Mean costs of delivering the SCRIPT intervention (pre- and post-test consultations, buccal kits and SNP test) and the control consultation, as well as health service utilisation and direct health care costs at 12 months
- Risk-appropriate screening behaviour after 5 years

### Participant timeline {13}

Table 1 shows the participant timeline.

**Table 1:** SCRIPT trial participant timeline

	Trial period						
	Enrolment	Allocation to intervention		Post-allocation^**a**^			
**Time point**	***0***	***0***	***2–3 weeks***	***1 month***	***6 months***	***12 months***	***5 years***
**Enrolment**							
**Eligibility screen**	X						
**Informed consent**	X						
**Allocation to intervention**		X					
**Interventions**							
***SCRIPT intervention***			X (PRS report)				
***Control***		X (cancer risk reduction brochure)				X (PRS report)	
**Assessments**							
***Demographics***	X						
***CRC screening + health service utilisation data (GP record, MBS, NBCSP, VAED)***						X	X
***CRC screening data (participant report)***	X			X	X	X	
***CRC risk perception***	X			X	X	X	
***Cancer-specific anxiety (Cancer Worry Scale)***	X			X	X	X	
***Influences of CRC screening behaviour (Preventative Health Model)***	X			X	X	X	
***Cancer screening intentions***	X			X	X	X	

^a^For the intervention arm, time points are after provision of PRS report and for the control arm, after standard cancer risk reduction information (i.e. last information regarding CRC risk and screening information)

### Sample size {14}

We used the results of the CRISP trial to inform our sample size calculations for this trial (paper under review, *Med. J. Aust.*). The original sample size of 218 participants for the grant application was based on a 20% difference between the intervention and control arms, assuming 25% in the control arm and 45% in the intervention arm, with 10% attrition at 12 months. However, in the CRISP trial, we found 40% had risk-appropriate screening in the control arm and 60% in the intervention arm of those due screening at 12 months for participants aged 50 years and older. The primary outcome had less than 5% missing data as it was derived from multiple data sources (such as GP record, Victorian Admitted Episodes Dataset (VAED), Medicare Benefits Schedule (MBS), the National Bowel Cancer Screening Program (NBCSP)) and did not rely only on self-report from a returned questionnaire. Furthermore, our original sample size calculation assumed all participants would be due a CRC screening test, as would be expected for participants aged above 50 years old based on current guidelines and the eligibility criteria. However, for participants aged 45 to 49 years, appropriate screening will be defined as no CRC screening if classified as having average CRC risk and iFOBT if they are classified as moderate CRC risk. Hence, to ensure that we had sufficient power to detect a difference appropriate CRC screening between the arms, we revised our sample size to allow for an attenuation in the intervention effect, explained as follows.

We estimated that 25% of the trial sample will be aged 45–49 years old, of which 90% will be at average risk and 10% at moderate risk [50]. We conservatively assumed that 95% of those aged 45 to 49 years in the average risk group will be appropriately screened in both trial arms (i.e. no CRC screening); those identified as moderate risk, we assumed that 70% in the intervention arm and 10% in the control arm would be appropriately screened (i.e. iFOBT screening). Thus, for those aged 45–49 years, the expected between-arm difference in appropriate screening would be 6% (92.5% intervention vs 86.5% in the control arm). We also assumed that for participants aged 50 years and older who were due a CRC screening test in the next 12 months, 60% would be appropriately screened in the intervention arm and 40% in the control arm (paper in submission).

Thus, we revised the sample size to detect a between-arm difference of 17% (68% vs 51%, after taking a weighted average of the proportions aged above or below 50 years). To detect this effect size, with 80% power and a two-sided 5% significance level, we require a total sample size of 274 participants at baseline (137 participants per arm), allowing for 5% of the primary outcome data to be missing.

This is an additional 56 participants to the trial from the original sample size calculations provided in the grant proposal and Trials registry. This sample size also provides 90% power (5% significance level) to detect a 20% between-arm difference for appropriate screening for participants aged 50 years and older (60% in the intervention and 40% in the control group, 5% missing data).

### Recruitment {15}

#### Identification of potential participants and recruitment

Patients aged between 45 and 70 on appointment lists of consented GPs within 1 week of the appointment will consecutively be approached. A consecutive approach is utilised to minimise selection bias and ensure a representative sample. The initial approach of potential participants by researchers will take place in one of two ways: face-to-face in the general practice waiting rooms or via telephone. In the era of COVID-19, community lockdowns have resulted in many GP appointments being moved to telehealth delivery and efforts being made to reduce waiting room traffic [51], we anticipate that eligible patients will mainly be approached via telephone.

#### Initial approach

Potential participants will be approached either in the waiting room or via telephone by a research assistant. Their eligibility will be checked, and they will be given an information sheet about the trial, either in hard copy or via email. Those approached via phone will be given the option to complete their recruitment in person or via teletrial.

#### Face-to-face recruitment

Interested potential participants with upcoming face-to-face appointments will be asked to attend their GP appointment 20 min early to meet with the researcher. Those whose appointments have already passed will be asked to attend their clinic at a convenient time. Confirmation of eligibility and obtaining informed consent in conducted in a private consulting room.

#### Teletrial recruitment

Interested potential participants who opt for teletrial recruitment, or those who cannot attend their clinic for research purposes due to COVID-19 lockdowns, will have the verbal portion of the consent completed over the phone. The study information sheet, consent forms, baseline questionnaire and DNA collection kit will then be posted to them. These participants are not considered enrolled in the trial until the consent form, baseline questionnaire and DNA sample are returned.

#### Ineligible patients and patients who do not wish to participate in the trial

An electronic recruitment log containing age and gender will be kept throughout recruitment. Reasons for ineligibility or refusal (if provided) will be recorded in REDCap. No identifying data will be kept for this group. This recruitment log will be maintained to track the representativeness of the recruited sample.

Two attempts will be made to reschedule non-attendances at recruitment appointments and to remind those who have received recruitment documents in the post, after which these potential participants will be marked as refused and their identifying information will be destroyed.

## Assignment of interventions: allocation

### Sequence generation {16a}

Participants will be randomly allocated 1:1 to the intervention or control arms. The allocation sequence will be computer-generated stratified by general practice using permuted blocks of random sizes. To ensure concealment, the block sizes will not be disclosed. Using restricted randomisation within the stratum ensures that the number of individuals is balanced between study arms.

### Concealment mechanism {16b}

The randomisation schedule will be embedded within the online database (REDCap [52]) which will automatically assign the participant after they complete the baseline survey to either the intervention or control arm ensuring allocation concealment. User permissions in REDCap will be restricted so that only the trial statistician has access to the schedule.

#### Implementation {16c}

A statistician not involved in the recruitment of participants or data collection will generate the randomisation schedule and upload it to the trial online database. Research assistants within the trial will enrol participants (defined as receipt of the study consent form, either in person or via post) and then randomise them using the randomisation module in the REDCap database.

## Assignment of interventions: blinding

### Who will be blinded {17a}

It is not possible to blind the participants or their GPs to their arm as they will receive their personal risk of CRC derived from the PRS if they are in the intervention arm. The control arm will receive standard cancer prevention information as an attention control and usual care from their GP with regard to their cancer screening.

Research staff who randomise the participant and return the PRS and screening recommendations to participants in the intervention arm also cannot be blinded. However, staff who conduct the questionnaire follow-up with the participants over the phone will be blinded. Staff who analyse the data will be blinded (arms will be designated randomly at that point as A and B).

### Procedure for unblinding if needed {17b}

As participants cannot be blinded and there must be some unblinded research staff to deliver the intervention to participants, there does not need to be a procedure for unblinding.

## Data collection and management

### Plans for assessment and collection of outcomes {18a}

#### Primary outcome

The primary outcome is the proportion of participants who have had risk-appropriate CRC screening at 12 months’ follow-up. The appropriate type and frequency of screen will be determined by participant’s level of CRC risk as follows:

For those aged 50 years and over:

- *Average-risk category*: for participants whose 10-year absolute risk of colorectal cancer is less than 4% based on their PRS, family history, age and sex (intervention only) and who do not fall into the moderate risk category of the 2017 NHMRC-endorsed Australian guidelines [5] (both trial arms), iFOBT every 2 years is considered risk-appropriate screening.
- *Moderate-risk category*: for participants whose 10-year absolute risk of colorectal cancer is greater than or equal to 4% based on their PRS, family history, age and sex (intervention only), or they had a self-reported family history of CRC (both trial arms) that places then in the moderate risk category of the 2017 Australian guidelines [5], risk-appropriate screening is colonoscopy every 5 years.

For those aged under 50 years:

- *Average-risk category*: for participants whose 10-year absolute risk of colorectal cancer is less than 1% based on their PRS, family history, age and sex (intervention only) and do not fall into the moderate risk category of the 2017 NHMRC-endorsed Australian guidelines [25] (both trial arms), risk-appropriate screening is no screening until aged 50 years.
- *Moderate-risk category*: for participants whose 10-year absolute risk of colorectal cancer is greater than or equal to 1% based on their PRS, family history, age and sex (intervention only), or they had a self-reported family history of CRC (both trial arms) that placed then in the moderate risk category of the 2017 Australian guidelines [5], risk-appropriate screening is iFOBT every 2 years until aged 50 years.

Exceptions to these rules are:

- *Moderate- or average-risk category*: If a participant had a history of colorectal adenomas or sessile serrated lesions, whether and when a colonoscopy should be repeated is based on the 2017 Australian guidelines [53] The timing and mode of follow-up test resulting from previous adenomas/sessile serrated lesions would take precedence over the screening recommended by standard national guidelines (control arm) or by the PRS (intervention arm).
- *Average-risk category and aged 50 years or over*: Those whose last test was a colonoscopy (for any reason), where the colonoscopy results do not require colonoscopic follow-up, are due an iFOBT after 4 years.

CRC screening behaviour will be collected via self-report by participants (via questionnaires at baseline, 1 month, 6 months and 12 months), as well as objective sources of data (GP record, Victorian Admitted Episodes Dataset (VAED), Medicare Benefits Schedule (MBS) and the National Bowel Cancer Screening Program (NBCSP)).

Screening prior to enrolment in the study as well as participant’s CRC risk category determines the timing of risk-appropriate screening within the 12-month follow-up period. All data sources with information on type of screening (self-report, GP record, VAED, MBS and NBCSP) will be used to determine screening prior to enrolment of participants. Self-reported events will be included, as this reflects what information a GP would use if considering screening to order in a standard consultation.

Objective data sources based on administrative data sources (GP record, VAED, MBS and NBCSP) will be used to determine whether a screening event has occurred within the 12-month follow-up period. Self-reported events will not be included, unless no objective data are available.

A Clinical Consensus Group (consisting of gastroenterologists and GPs, blinded to trial arm allocation) will review participants with complex past histories and use all available data sources to determine what risk-appropriate screening would be consistent with NHMRC surveillance and screening guidelines.

#### Secondary outcomes

Data for secondary outcomes will be collected primarily from participants’ questionnaires at 1, 6 and 12 months post-intervention delivery.

- Participants will be asked to state their CRC risk perception numerically, on a scale from 0 to 100, and comparatively to an ‘average’ person, from 1 — much lower — to 7 — much higher [46].
- The Cancer Worry Scale [47] is a validated, 6-item scale measuring anxiety about the risk of CRC. Each item is rated on a 1–4 scale (not at all or rarely to almost all the time) and then summed for a total.
- The Preventative Health Model [48] is an 18-item, validated scale, answered on a five-point Likert scale from strongly disagree to strongly agree. It measures known predictors of CRC screening behaviour in five subscales: salience and coherence (4 items), response efficacy (2 items), cancer worries (2 items), social influence (4 items) and self-efficacy (6 items). Each subscale is summed for a total.
- CRC screening intentions to undertake screening or discuss screening with their GP will be measured with a five-point Likert scale from strongly disagree to strongly agree.
- The cost of the SCRIPT intervention, health service utilisation and health care costs will be calculated based on the assessment of GP consultations, colonoscopy services, iFOBT, and associated pathology services obtained from audit of GP records, and data from the MBS, VAED and NBCSP records. Any other associated changes in health care utilisation will be captured through access to participants’ MBS and GP record data. Indirect costs will not be included.
- Five-year impact on risk-appropriate screening behaviour and health service utilisation will be measured but only utilising objective, administrative data. Impact on screening behaviour will be assessed as per the primary outcome.

### Plans to promote participant retention and complete follow-up {18b}

Prior to consent, participants will be advised that they can withdraw from the study at any time without giving a reason. If they withdraw, there will be no further contact from the study, including follow-up questionnaires. Any data already collected will be included in analyses and objective screening data will still be collected as this does not require contact with the participant. Participants who withdraw can also choose to withdraw all their data that they have previously provided, except when their data have been processed and analysed. If this is the case, any unprocessed data that has not been included in an analysis at the time of withdrawal will be destroyed and their data excluded from the analysis.

Reminders will be made to participants regarding each follow-up questionnaire. Telephone contact will initially be attempted, with the offer to complete the questionnaire over the phone. If the participant cannot be contacted via phone, email and/or SMS reminders will be sent. A maximum of two reminders (i.e. successful contact via phone or sending of an email or SMS) will be made; then, this questionnaire is considered lost to follow-up. Participants will still be sent subsequent questionnaires unless explicitly withdrawing from the trial.

### Data management {19}

Data will be collected, managed and stored according to the study’s data management plan, developed in accordance with the University of Melbourne’s Research Data Management Policy and Research Code of Conduct. A REDCap database will be used to collect and store data, only accessible by authorised and trained researchers. REDCap is a password-protected online database. The REDCap database has mandatory data entry fields to reduce missing data, range checks for the data values and branching questions. Before randomisation, REDCap provides a pop-up for researchers to doublecheck data entry of the stratifying variable. All paper-based data will be entered directly into REDCap and these will be stored securely in an office within the Victorian Comprehensive Cancer Centre in a locked file cabinet. All data will only be accessible to researchers listed on ethical approvals.

### Confidentiality {27}

Prior to consent, any information about potential participants will not leave the patient’s general practice and is not retained by researchers.

In accordance with the University of Melbourne’s Research Data Management Policy and Research Code of Conduct, participants’ research data will be stored on University managed and/or sanctioned storage infrastructure. Data will be secured via a personal login and data elements restricted by role at the direction of the chief investigator. Participant contact information (address, phone number and email address) will be stored on REDCap only, and access will be restricted to members of the research team who require it for study-related contact. This restriction will be built into REDCap user roles. Each participant will be given a unique de-identified study code and all data extracted from the REDCap database will only contain this study code. Paper-based data will be destroyed using confidential waste management services 5 years after the publication of the results.

Individual-level data will be made available to external researchers on a case-by-case basis. This excludes data provided by external organisations (Services Australia (MBS data), AIHW (NBCSP data) and DHHS (VAED data)), as well as data collected from GP records. It also excludes identifying data and only age brackets rather than dates of birth will be provided. Sharing of this data will only take place after ethical approval.

### Plans for collection, laboratory evaluation and storage of biological specimens for genetic or molecular analysis in this trial/future use {33}

DNA will be collected with ORAcollect®-DNA OCR-100 saliva collection kits (DNA Genotek, Ottawa, ON, Canada). Genotyping will be conducted by Genetic Technologies using their GeneType for Colorectal Cancer test (https://www.gtglabs.com/colorectal-cancer). DNA samples will be processed and genotyped according to their National Association of Testing Authorities (NATA)-approved processes and any excess DNA disposed of according to the same guidelines. Participant DNA will not be retained for future use.

## Statistical methods

### Statistical methods for primary and secondary outcomes {20a}

The primary analysis will be based on the intention to treat principle where all randomised participants will be analysed in their allocated study arm. Baseline characteristics of the two arms will be summarised using descriptive statistics. Possible differential attrition will be assessed by comparing baseline characteristics of those who withdraw against those who remain in the study. For the primary outcome analysis, a generalised linear model with the identity link function and binomial family will be used to estimate the absolute difference in the proportion of participants who have had risk-appropriate CRC at 12 months between intervention and control arms. We will also estimate the odds ratio using logistic regression. In all regression analysis, the randomisation stratification factor, general practice, will be included as fixed effect. The absolute (between-arm difference in the proportions) and relative (odds ratio) estimated effect sizes will be presented with their respective 95% confidence interval (CI), and the *p*-value will be estimated using logistic regression. Comparisons between arms on continuous secondary endpoints will be undertaken using a linear mixed-effects model that includes arm (intervention and control), general practice and time (baseline, 1, 6 and 12 months) as fixed effects and individuals as random effect, with two-way interaction between arm and time, except baseline where study arm means will be constrained to be equal. Differences between arms on binary secondary endpoints will be estimated using logistic regression using generalised estimating equations with robust standard errors to allow for the repeated outcome measures on individuals (1, 6 and 12 months), adjusting for general practice. All analyses will be conducted in Stata 17 [54].

The health economic analysis combines a within-trial and modelled analysis of the cost-effectiveness of implementing the genomic test. The within-trial analysis will consider the costs per appropriately screened individual in the two arms. The modelled analysis will consider how the incremental cost per appropriately screened individual might vary given differences in population incidence of the SNP variants, as well as potential variations in test sensitivity/specificity, and compliance with screening recommendations. Within-trial costs will be estimated based on data obtained from general practice, Medicare, NBCSP and VAED records. Mean estimates of costs will be used, and confidence intervals will be generated by resampling (bootstrap) techniques. Results for both analyses will be presented in terms of an incremental cost-effectiveness ratio as the cost per risk-appropriate screened participant.

### Interim analyses {21b}

No interim analyses are planned.

### Methods for additional analyses (e.g. subgroup analyses) {20b}

Sensitivity analyses will adjust for pre-specified baseline variables, such as risk group at baseline and whether recruitment occurred in person or via telehealth, in the regression models for the primary and secondary outcomes.

Sensitivity analyses are also planned to explore the impact on the intervention effect when using different data sources to define the primary outcome. For instance, defining risk-appropriate screening based on information from participant self-report and GP records on past screening at baseline (e.g. previous findings at colonoscopy, date of the last iFOBT). This is to emulate the real-world setting where the GP would not have access to additional administrative data during the consultation to determine what type of CRC screening was due (MBS, VAED). Sensitivity analyses will also test the robustness of the result to variations in the underlying assumptions and inputs to the health economic analysis.

Further additional analyses, including sensitivity, subgroup and supplementary analyses, will be described in a detailed statistical analysis plan (SAP) that will be made available prior to the analysis of the primary outcome.

### Methods in analysis to handle protocol non-adherence and any statistical methods to handle missing data {20c}

We will perform an adherence-adjusted analysis to account for non-compliance with uptake of the genomic test, as well as non-complete CRC risk result appointments, for the primary outcome [51, 55]. Appropriate methods for dealing with missing endpoint data will be undertaken, informed by a blinded review of the data. Details for the adherence-adjusted analyses and statistical methods to handle missing data will be provided in the SAP.

### Plans to give access to the full protocol, participant-level data and statistical code {31c}

Only researchers who require it will have access to the participant-level dataset stored in REDCap. All authors will have access to the full protocol. To assist with reproducible research, the statistical code will be made available to other researchers upon reasonable request.

## Oversight and monitoring

### Composition of the coordinating centre and trial steering committee {5d}

#### Coordinating centre

The coordinating centre will be responsible for the day-to-day running of the trial (SS, LB, MM, MK, SM, JMc and JE). The trial coordinator (SS) and chief investigator (JE) will oversee the running of the trial, including governance and administrative responsibilities (e.g. ethical approvals and maintenance of the protocol), identification and approach of general practices, GP and participant recruitment, data collection, verification and management, randomisation, delivery of the intervention and maintenance of the study budget. Additionally, the coordinating centre will organise steering committee meetings, draft study reports for the funding body and draft manuscripts.

#### Trial steering committee

A trial steering committee (SS, PC, SM, RDAL, MC, GF, JMa, CO, FW, DB, IW, JMc and JE) has been established to provide expert advice and oversight and ensure that the trial is conducted to the required standards. The steering committee includes the Chief Investigators, Associate Investigators and senior researchers from the coordinating centre. The steering committee is responsible for agreement of final protocol, protocol changes and reviewing progress throughout the study.

### Composition of the data monitoring committee, its role and reporting structure {21a}

This trial provides a personalised risk of CRC and screening recommendations to participants; any final decisions regarding CRC screening will be left to the participant in discussion with their GP. The intervention itself therefore is relatively low risk. This is a relatively small phase II efficacy trial. We do not expect significant adverse effects arising from the trial itself; there is no evidence that returning genomic risk results in substantial psychological distress [44]. We have therefore decided not to have a separate data monitoring committee. Oversight of the trial will be managed by the trial steering committee.

### Adverse event reporting and harms {22}

All protocol deviations will be recorded in the participant record and reported to the study coordinator and lead investigator (SS and JE), who will assess for seriousness.

Those deviations deemed to affect to a significant degree the rights of a trial participant or the reliability and robustness of the data generated in the clinical trial will be reported as serious breaches. Reporting will be done in a timely manner (within 72 h to the study coordinator and lead investigator) and within 7 days to the site’s Research Governance Office. The study coordinator and lead investigator must review and report serious breaches to the approving HREC within 7 days.

Where non-compliance significantly affects participant protection or reliability of results, a root cause analysis will be undertaken, and a corrective and preventative action plan prepared.

Where protocol deviations or serious breaches identify protocol-related issues, the protocol will be reviewed and, where indicated, amended.

### Frequency and plans for auditing trial conduct {23}

Researchers in the coordinating centre will meet at least weekly with the principal investigator to discuss and review trial progress. The principal investigator is contactable for prompt reporting of adverse events. The steering committee will meet quarterly, with more frequent meetings added as required throughout the duration of the trial set-up, recruitment and post-recruitment analysis phase. Minutes of all meetings will be digitally stored with all trial documentation. Progress will be reported to the trial funder every 12 months. There will be no independent auditing of trial conduct.

### Plans for communicating important protocol amendments to relevant parties (e.g. trial participants, ethical committees) {25}

This trial will be conducted in compliance with the current version of the protocol. Any change to the protocol document or informed consent form that affects the scientific intent, trial design and participant safety or may affect a participant’s willingness to continue participation in the trial is considered an amendment and therefore will be written and filed as an amendment to this protocol and/or informed consent form. All such amendments will be submitted to the HREC, for approval prior to being implemented.

### Dissemination plans {31a}

Data from this trial will be disseminated in several ways. Informal dissemination of results will occur with participants, participating GPs and other collaborators. Participants in the study are given the option at the time of consent to receive a plain language, one-page summary of the study findings after analysis is completed. Other collaborators will receive a similar summary, tailored to their position and interests (i.e. consumers will receive a lay summary).

Results of this research will be published in peer-reviewed journals. Additionally, the Primary Care Collaborative Cancer Clinical Trials Group (PC4) (who supports the study) facilitate communication and dissemination to a wide audience, including media releases to health professional and general outlets; Twitter and other social media outlets; PC4 Research Round-up and other health professional and general podcasts; and dissemination via the PC4 Consumer Advisory Group and their respective consumer networks. We will use all these approaches to promote the trial results. The chief investigators of the study hold primary responsibility for publications of results of the trial.

## Discussion

This trial aims to determine whether a personalised CRC risk estimate derived from a PRS with tailored screening recommendations and delivered in primary care increases risk-appropriate CRC screening. Precision cancer screening aims to recommend the most appropriate type and timing of screening based on risk but will not result in efficiency gains and reduction of mortality and morbidity from disease unless patients adhere to these recommendations.

Since the study began recruitment in April 2021 in Melbourne, Australia, our city has undergone several strict lockdowns due to the COVID-19 pandemic. Throughout the protocol described thus far, several contingencies have been outlined in our processes to reduce or remove the need for research staff to travel to general practices during recruitment. Some of these contingencies were planned before recruitment began, for example provisions for teletrial recruitment. Some, however, were proposed and implemented after recruitment began when lockdowns became lengthy, namely discussion of PRS results and screening recommendations for the intervention arm via teletrial rather than in person. All elements of this part of the intervention were replicated as closely as possible in the teletrial version, e.g. visually guiding participants through their PRS risk report using share screen facilities in teletrial software. This protocol amendment was discussed and approved by our trial steering committee including the trial statistician.

We considered carefully whether to employ a cluster randomised or individually randomised design for this trial. Cluster randomisation has the advantage of reducing the risk of contamination in the control arm but requires substantially larger sample sizes and may be subject to selection bias [56]. When clusters are of a moderate size and the risk of contamination is low, individual randomisation may be utilised [57]. Our previous individually randomised trial with a similar design, recruitment method and outcomes to this trial showed a low level of contamination (paper in submission). Consequently, we decided to randomise at an individual level in this trial.

A considerable point of discussion amongst the trial steering committee was the exclusion criteria for those who have at least one grandparent born in Africa. The scientific justification for this criterion is outlined above, as are the alternatives that we incorporated into the study protocol for those who met this exclusion criterion. There were several aspects that the steering committee considered when making this judgement. Firstly was the poorer accuracy of PRSs in those of African ancestry [30, 58]. Secondly was the ethical consideration that precision screening ultimately aims to increase equitable access to the best form of screening for an individual’s risk and providing access to a risk stratifying algorithm only for a portion of the population is likely to do the opposite. Thirdly was the already lower uptake of CRC screening for disadvantaged segments of the Australian population, including those who are culturally and linguistically diverse [17]. After careful consideration by the trial steering committee, we felt the risk of providing an inaccurate risk estimate to those of African ancestry was too great but were able to offer an alternative, more accurate phenotypic method of risk assessment to this group outside the trial, in collaboration with their GP.

The evidence behind PRSs to predict the risk of CRC is constantly growing [11, 59]. There are now approximately 100 SNPs associated with the risk of CRC [11]. These newer PRSs with more SNPs included show marginally better discrimination between cases and controls and ability to separate risk in population than those scores with fewer SNPs [8, 11]. As this evidence evolves, and newer PRSs become available with sufficient validation, as well as logistically available to be offered within the study, the study investigators may update the PRS in the study to be in line with the latest evidence for later participants. We believe this reflects likely future models of updating PRSs in clinical service; furthermore, given that the primary outcome is a behavioural one and not one based on cancer incidence, there are no methodological implications of using the most up-to-date PRS if feasible to do so.

Due to lengthy delays in approvals from Services Australia for the trial to include collection of MBS data from participants, the trial steering committee decided that recruitment should begin before this approval was granted. This resulted in the first study participants not providing written consent for the release of their MBS data at the time of recruitment. Approvals have now been granted from Services Australia and these initial participants are being recontacted for the purposes of this consent. However, this may result in some participants not being consented to access their MBS data (private colonoscopies and iFOBT kits ordered by GPs outside the NBCSP). The risk is that there may be screening events for some participants not captured in any other data source (GP records, VAED and NBCSP) which would alter the assessment of their primary outcome. However, in our experience of a previous trial using similar methods (paper in submission), there is sizeable overlap between information about colonoscopies and GP-ordered iFOBT captured in MBS, VAED and GP records. Therefore, we anticipate that the overall risk of bias in determining the primary outcome due to this issue is low.

This trial includes secondary outcomes that allow us to examine the effect of the intervention on behavioural predictors of CRC screening; this is particularly important should we not observe a clinically important difference in the proportion of risk-appropriate screening between arms. These secondary outcomes were selected from our pilot and development work of the intervention [39, 60], based on health behaviour theories [49, 61]. They include elements to determine whether participants’ attitudes towards screening were altered by the intervention, their perception of their own risk of CRC, the impact of risk prediction on their cancer worry and whether any non-adherence to screening recommendations can be attributed to GP behaviour rather than patient behaviour (e.g. if participants were not referred for discussion of colonoscopy or an iFOBT was not ordered by the GP, as determined by audit of GP records). These secondary outcomes will also provide valuable data to incorporate into a GP implementation strategy should the trial show a positive impact on risk-appropriate screening behaviour, allowing this strategy to focus on elements of the intervention that are proposed to have the greatest behavioural effect.

This study will test whether delivery of a CRC PRS in primary care is effective in encouraging risk-appropriate CRC screening compared to standard cancer risk reduction information in patients attending general practice. CRC population screening targeted using a PRS has the potential to not only recommend the most appropriate screening for an individual’s risk, but to encourage them to complete that screening. This would result in a cost-effective targeted screening programme, with the potential to reduce morbidity and mortality from CRC. If precision CRC screening is to be implemented across the population, we must determine how and where risk assessment will occur and whether the public are likely to accept and adhere to this new way of recommending screening. This study will provide valuable data regarding whether this could potentially be a cost-effective approach delivered in primary care, as the first point of health care provision for the vast majority of Australians.

### Trial status

Protocol version 1.2, 14 February 2022. Trial recruitment began on 19 April 2021. Trial recruitment is estimated to be completed in October 2022.

## Supplementary Information

**Additional file 1.** Standard cancer risk reduction information provided to control participants in the SCRIPT study.

**Additional file 2.** Example colorectal cancer risk report and screening recommendations for a participant’s GP at moderate risk in the SCRIPT study.

**Additional file 3.** Example colorectal cancer risk report and screening recommendations for a participant’s GP at moderate risk in the SCRIPT study.

**Additional file 4.** SCRIPT study consent form.

## Data Availability

SS and PC will have access to the final trial dataset. Non-identifiable data may be provided on request of external researchers after publishing the findings. The steering committee will manage external requests for data.

## Abbreviations

ANZCTR: Australian New Zealand Clinical Trials Registry
AUROC: Area under the receiver operating curve
CRC: Colorectal cancer
CRISP: Colorectal cancer RISk Prediction tool
FIT: Faecal immunochemical test
FOBT/iFOBT: (Immunochemical) faecal occult blood testing
GP: General practitioner
GWAS: Genome-wide association study
MBS: Medicare Benefits Schedule
NBCSP: National Bowel Cancer Screening Program
NHMRC: National Health and Medical Research Council
PC4: Primary Care Collaborative Cancer Clinical Trials Group
PRS: Polygenic risk score
RA: Research assistant
RACGP: Royal Australian College of General Practitioners
RCT: Randomised controlled trial
SCRIPT: SNP Cancer Risk Prediction Trial
VAED: Victorian Admitted Episodes Data
