# Supplementary material for: The SCRIPT trial: study protocol for a randomised controlled trial of a polygenic risk score to tailor colorectal cancer screening in primary care
Source: Trials. 2022 Sep 27;23:810. doi: 10.1186/s13063-022-06734-7 (PMC9513012; doi:10.1186/s13063-022-06734-7)
Supplement: Supplementary file 1 — Additional file 1. Standard cancer risk reduction information provided to control participants in the SCRIPT study. [file 13063_2022_6734_MOESM1_ESM.pdf]

CUT YOUR CANCER RISK

BE PHYSICALLY ACTIVE

1 hour of moderate activity or 30 minutes of vigorous activity daily is recommended

MAINTAIN A HEALTHY WEIGHT

A waistline > 94cm in men and 85cm in women increases the risk of some cancers

Balance what you eat with your physical activity to keep your weight down

LIMIT ALCOHOL

No more than 2 standard drinks a day and at least 1 or 2 alcohol free days per week

EAT A HEALTHY DIET

Eat 5 serves of vegetables and 2 serves of fruit every day

Limit red meat to 3 - 4 serves a week

Limit/avoid eating processed meat: bacon, sausages, salami, ham etc

QUIT SMOKING

Call the Quitline on **13 78 48** or visit **www.quit.org.au**
