# Supplementary material for: The SCRIPT trial: study protocol for a randomised controlled trial of a polygenic risk score to tailor colorectal cancer screening in primary care
Source: Trials. 2022 Sep 27;23:810. doi: 10.1186/s13063-022-06734-7 (PMC9513012; doi:10.1186/s13063-022-06734-7)
Supplement: Supplementary file 2 — Additional file 2. Example colorectal cancer risk report and screening recommendations for a participant’s GP at moderate risk in the SCRIPT study. [file 13063_2022_6734_MOESM2_ESM.pdf]

SCRIPT TRIAL

# GP REPORT



Patient: Participant A

Date of Birth: 01-01-1965

Australian Government
National Health and Medical Research Council

Based on the National Health Guidelines (NHMRC)[1] and the patient's bowel cancer risk, the patient is **recommended to have a faecal occult blood test (FOBT) every 2 years.**

**The patient's absolute risk of developing bowel cancer in the next 10 years is** **1.03%**

This risk was calculated from their DNA test, their family history of bowel cancer, their sex and their age.

**Important:**

**A colonoscopy is not recommended for the patient based on this assessment.**

**Colonoscopies have potential for harm including a risk of causing bleeding, bowel perforation and death.**

1. Cancer Council Australia Colorectal Cancer Guidelines Working Party. Clinical practice guidelines for the prevention, early detection and management of colorectal cancer. Sydney: Cancer Council Australia 2018

## FURTHER INFORMATION

For further information please contact:

**Sibel Saya : Study Coordinator & Genetic Counsellor**

**P:** xx xxxx xxxx **or E: script-trial@unimelb.edu.au**

THE UNIVERSITY OF MELBOURNE

Victorian Comprehensive Cancer Centre
University of Melbourne, Level 10,
305 Grattan Street, Melbourne 3010

**Ethics ID:** 2057592.1

SCRIPT TRIAL

# GP REPORT



Patient: Participant A

Date of Birth: 01-01-1965

This diagram shows what would happen to 100,000 people like your patient if they have either **a faecal occult blood test (FOBT)**, **no bowel cancer screening** or **a colonoscopy.**

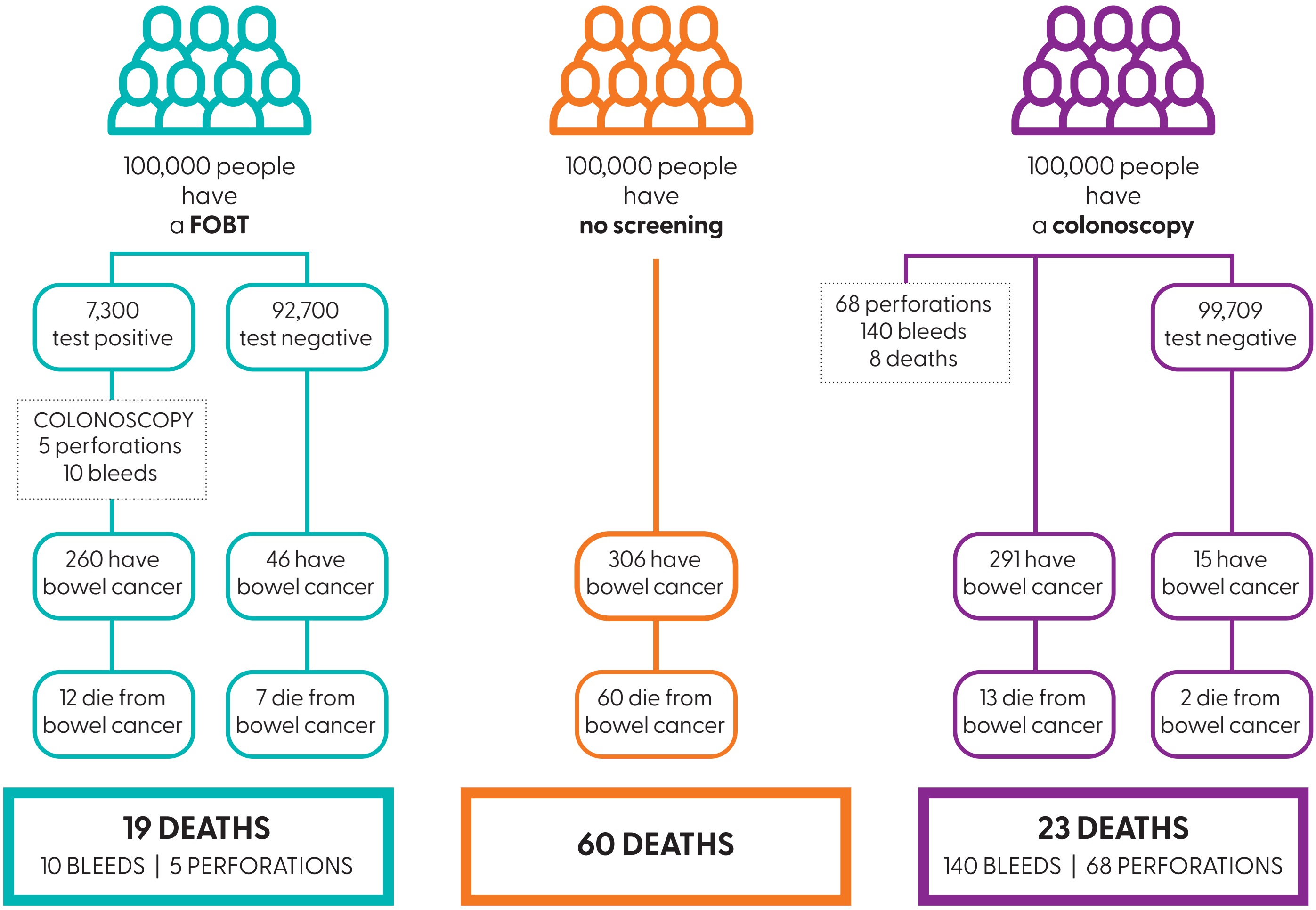


The National Bowel Cancer Screening Program will send this patient a bowel screening kit **every 2 years until they are 74.**

These recommendations are based on the current best information about the impact of DNA variation on bowel cancer risk and the patient's family history. If their family history of cancer changes, their screening recommendations might need review.
