# Supplementary material for: The SCRIPT trial: study protocol for a randomised controlled trial of a polygenic risk score to tailor colorectal cancer screening in primary care
Source: Trials. 2022 Sep 27;23:810. doi: 10.1186/s13063-022-06734-7 (PMC9513012; doi:10.1186/s13063-022-06734-7)
Supplement: Supplementary file 3 — Additional file 3. Example colorectal cancer risk report and screening recommendations for a participant’s GP at moderate risk in the SCRIPT study. [file 13063_2022_6734_MOESM3_ESM.pdf]

SCRIPT TRIAL

GP REPORT

Patient: Participant B

Date of Birth: 01-01-1960

Australian Government
National Health and Medical Research Council

Based on the National Health Guidelines (NHMRC)[1] and the patient's risk of developing bowel cancer in the next 10 years, they are **recommended to have a colonoscopy every 5 years.**

The patient's absolute risk of developing bowel cancer in the next 10 years is **5.3%**

This risk was calculated from their DNA test, their family history of bowel cancer, their sex and their age.

1. Cancer Council Australia Colorectal Cancer Guidelines Working Party. Clinical practice guidelines for the prevention, early detection and management of colorectal cancer. Sydney: Cancer Council Australia 2018

This graph shows the patient's **risk of developing bowel cancer over time,** compared to the risk of the general Australian population. These recommendations are based on the current best information about the impact of DNA variation on bowel cancer risk and their family history. If their family history of cancer changes, their screening recommendations might need review.

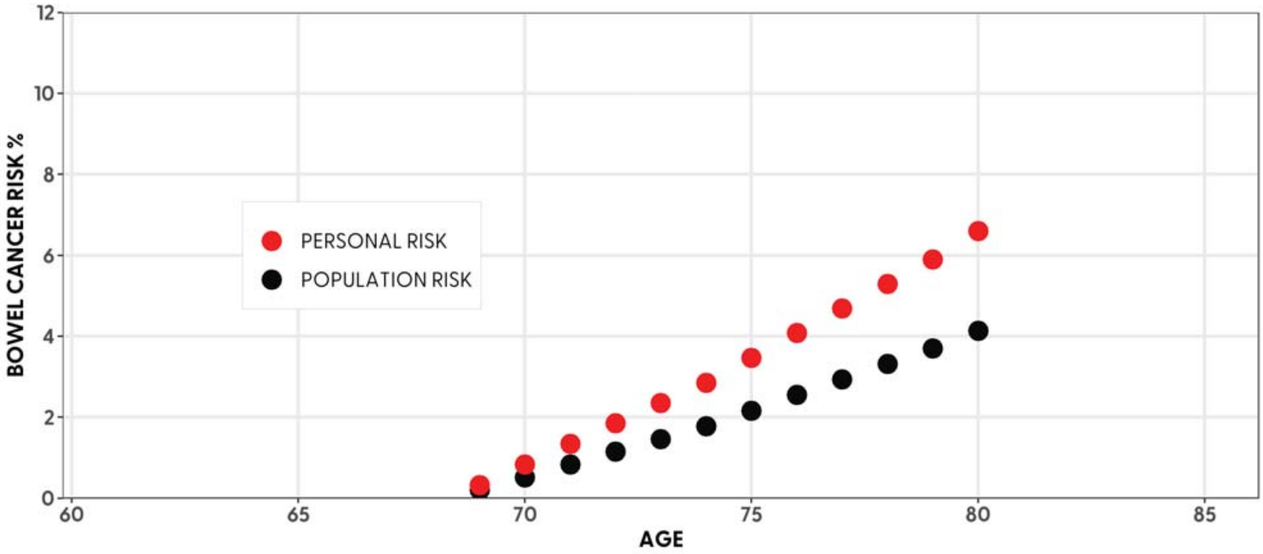

SCRIPT TRIAL

GP REPORT

Patient: Participant B

Date of Birth: 01-01-1960

This diagram shows what would happen to 100,000 people like your patient if they have either **a faecal occult blood test (FOBT)**, **no bowel cancer screening** or **a colonoscopy.**

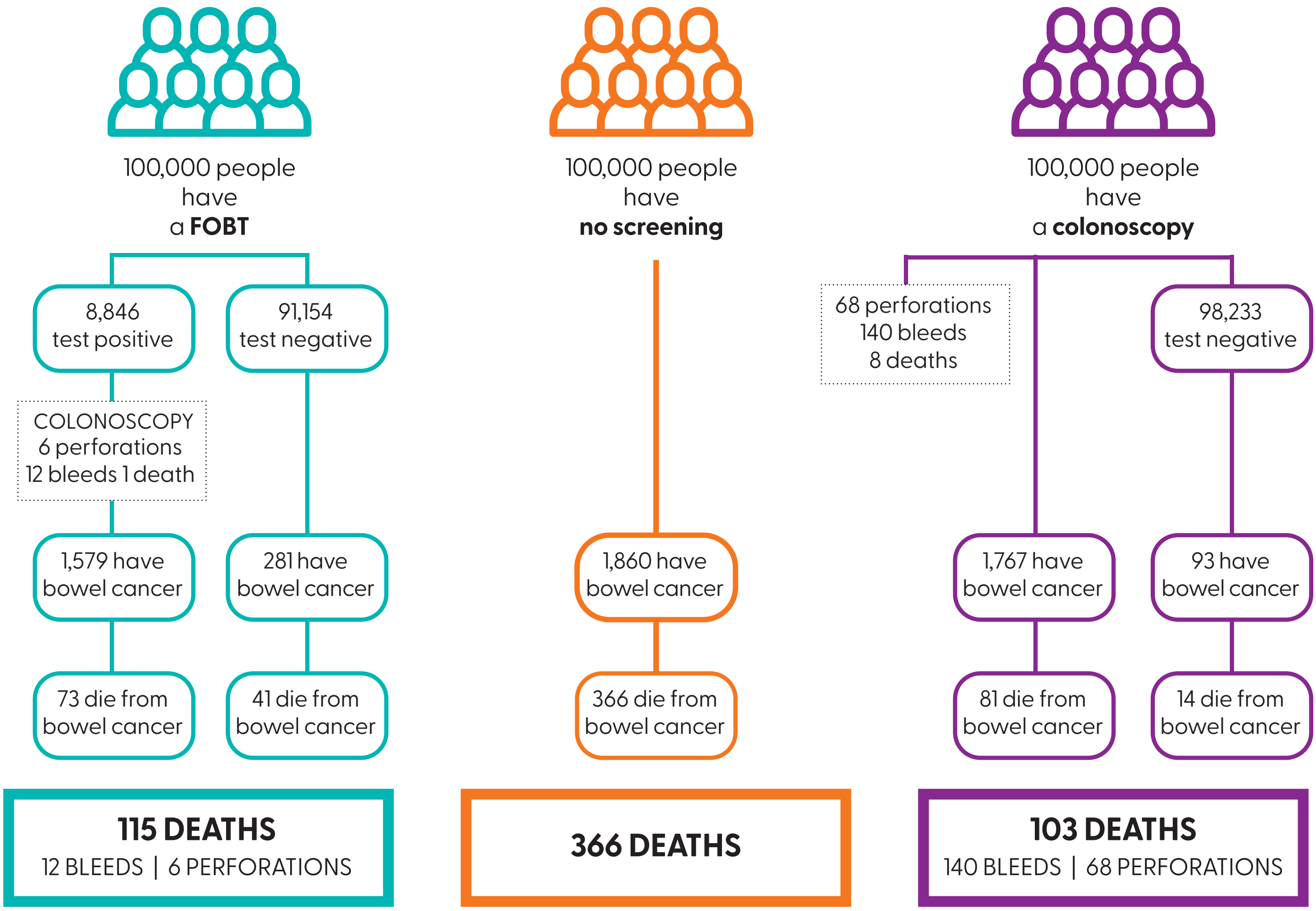

These recommendations are based on the current best information about the impact of DNA variation on bowel cancer risk and the patient's family history. If their family history of cancer changes, their screening recommendations might need review.

FURTHER INFORMATION

For further information please contact:

Sibel Saya : Study Coordinator & Genetic Counsellor
P: xx xxxx xxxx **or E: script-trial@unimelb.edu.au**

THE UNIVERSITY OF MELBOURNE

Victorian Comprehensive Cancer Centre
University of Melbourne, Level 10,
305 Grattan Street, Melbourne 3010

Ethics ID: 2057592.1
