# Supplementary material for: The SCRIPT trial: study protocol for a randomised controlled trial of a polygenic risk score to tailor colorectal cancer screening in primary care
Source: Trials. 2022 Sep 27;23:810. doi: 10.1186/s13063-022-06734-7 (PMC9513012; doi:10.1186/s13063-022-06734-7)
Supplement: Supplementary file 4 — Additional file 4. SCRIPT study consent form. [file 13063_2022_6734_MOESM4_ESM.pdf]

THE UNIVERSITY OF MELBOURNE

ID: __ __ __/__ __ __

Centre for Cancer Research & Department of General Practice
The University of Melbourne

PATIENT PARTICIPANT CONSENT FORM

The SCRIPT Trial: a study of DNA testing to tailor bowel cancer screening in primary care

Investigators:

Prof Jon Emery, Prof Mark Jenkins, Prof Finlay Macrae, Prof Ingrid Winship, Ms Sibel Saya, Dr Dan Buchanan, Dr Fiona Walter, Dr Patty Chondros, Dr Richard De Abreu Lourenco, Dr Jenny McIntosh

---

1. I consent to take part in this research project, the details of which have been explained to me, and I have been provided with a written participant information brochure form (Patient PLS, V1) to keep.
2. I understand that after I sign and return this consent form it will be retained by the researcher.
3. I understand participation in this study will involve a DNA test via a saliva collection tube for bowel cancer risk. I understand if I am randomly allocated to the intervention group, I will receive results of my DNA test about my future risk of bowel cancer and advice about bowel cancer screening.
4. I understand that if I am randomly allocated to the control group, I can receive the results of my DNA test at the end of the study period (approximately 12 months).
5. I understand my participation will involve completing three questionnaires over the next 12 months.
6. I agree that the researchers may use the results as described in the participant information brochure.
7. I agree to the release of my medical information from my medical records including general practice, Medicare, the National Bowel Cancer Screening Program and Victorian Hospital data (Victorian Admitted Episodes Dataset, VAED) that relates only to screening for bowel cancer for the past five years and next five years.
    a. I understand that I will be asked to sign a consent form authorising the study to access my complete Medicare Benefits Schedule (MBS) data as outlined in the consent form. Medicare collects information on my doctor visits and the associated costs. The consent form is then sent securely to Services Australia who holds MBS data confidentially.
8. I consent to the research team contacting me for a follow up interview about my experience of being involved in the study. The interview will be recorded. It is optional to participate.
9. I acknowledge that:
    a. The project is for the purposes of research;
    b. The possible effects of participating in this study have been explained to my satisfaction;
    c. I understand that my involvement in this project is entirely voluntary;
    d. I have been informed that I am free to withdraw from the project at any time without explanation or prejudice and to withdraw any unprocessed data I have provided;
    e. I have been informed that the confidentiality of the information and samples I provide will be safeguarded subject to any legal requirements;
    f. I have been informed that my consent form and information will be stored securely at the

University of Melbourne and securely destroyed 5 years after publication of the results;

g. My name will not be identified in any publications arising from this research project;

h. In accordance with the law of Victoria, I understand that it is possible for data to be subject to subpoena, or freedom of information request.

10. I understand that I can withdraw from the study and are under no obligation to continue with the research study. I may change my mind at any time about participating in the research. People withdraw from studies for various reasons and I do not need to provide a reason. I can withdraw from the study at any time by completing and signing the 'Participant Withdrawal of Consent Form' which has been provided to me, and is to be completed by me and supplied to the research team if I choose to withdraw at a later date. If I withdraw from the study, I will be able to choose whether the study will destroy or retain the unprocessed information it has collected about me. I should only choose one of these options. Where both boxes are ticked in error or neither box is ticked, the study will destroy all unprocessed information it has collected about me.

11. I understand my consultation might be audio-recorded and that this is voluntary. The recording will only be used for research purposes and my choice not to be audio-recorded will not be disclosed to anyone except the research team.

Please tick one box: ☐ I consent to be recorded

☐ I do not consent to be recorded

12. I agree to be approached about potential involvement in future studies led by the Cancer in Primary Care Research Group at the University of Melbourne. I will be under no obligation to participate in these future studies.

Please tick one box: ☐ I give consent to be approached about future studies

☐ I do not consent to be approached about future studies

Name of participant: ____

Participant signature: ____________________________________ Date: ___________

Researcher's Name (printed) ____

Signature: ____ Date: __________

At the conclusion of this research project, I would like to receive a copy of the results summary;
Please tick one box: ☐ Yes ☐ No
If **yes**, please write the email address you would like this sent to:
E-mail: ____

If you have a privacy complaint in relation to the use of your MBS data you should contact the Office of the Australian Information Commissioner. You will be able to lodge a complaint with them.

Website: www.oaic.gov.au | Telephone: 1300 363 992 |Email: enquiries@oaic.gov.au

Mail: GPO Box 5218, Sydney NSW 2001
